# Supplementary material for: Structural Elucidation of a Glucan from Trichaster palmiferus by Its Degraded Products and Preparation of Its Sulfated Derivative as an Anticoagulant
Source: Mar Drugs. 2023 Feb 24;21(3):148. doi: 10.3390/md21030148 (PMC10056992; doi:10.3390/md21030148)
Supplement: Supplementary file 1 [file marinedrugs-21-00148-s001.zip › marinedrugs-2217221-supplementary.pdf]

Supplementary data

Structural elucidation of a glucan from *Trichaster palmiferus* by its degraded products and preparation of its sulfated derivative as an anticoagulant

Haiqiong Ma [1], Qingxia Yuan [1,2,*], Hao Tang [1], Hongjie Tan [1], Tingting Li [1], Shiying Wei [1], Jinwen Huang [1], Yue Yao [1], Yaping Hu [1], Shengping Zhong [1,2], Yonghong Liu [1,2], Chenghai Gao [1,2,*] and Longyan Zhao [1,2,*]

[1] Institute of Marine Drugs, Guangxi University of Chinese Medicine, Nanning, 530200, China

[2] Guangxi Key Laboratory of Marine Drugs, Guangxi University of Chinese Medicine, Nanning, 530200, China

* Correspondence: qingxiayuan@163.com (Q.Y.); gaochh@gxtcmu.edu.cn (C.G.); longyanzhao@gmail.com (L.Z.)

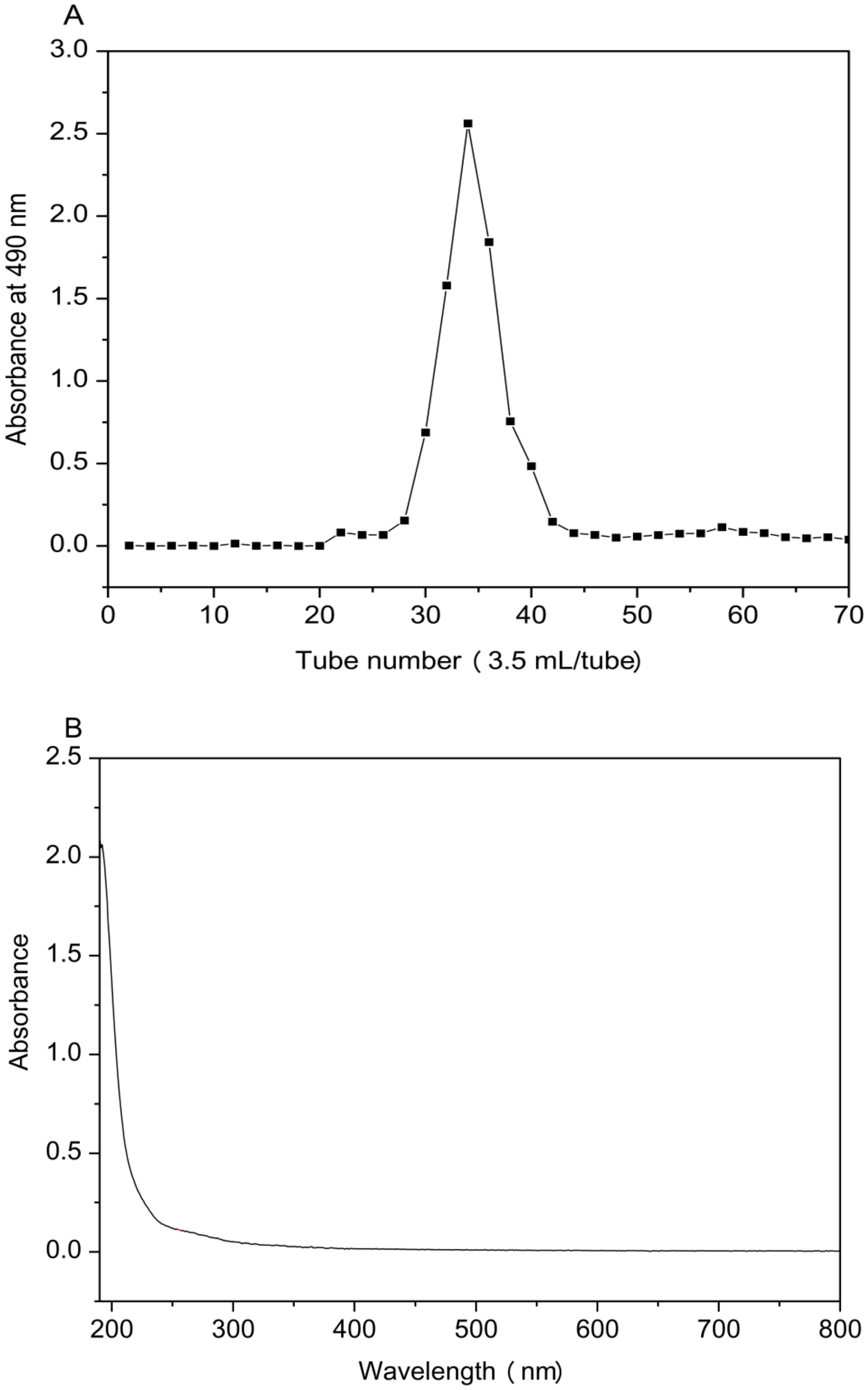

Figure S1. Elution profile of TPG on a Sepharose CL-6B column (A) and its UV spectrum (B)

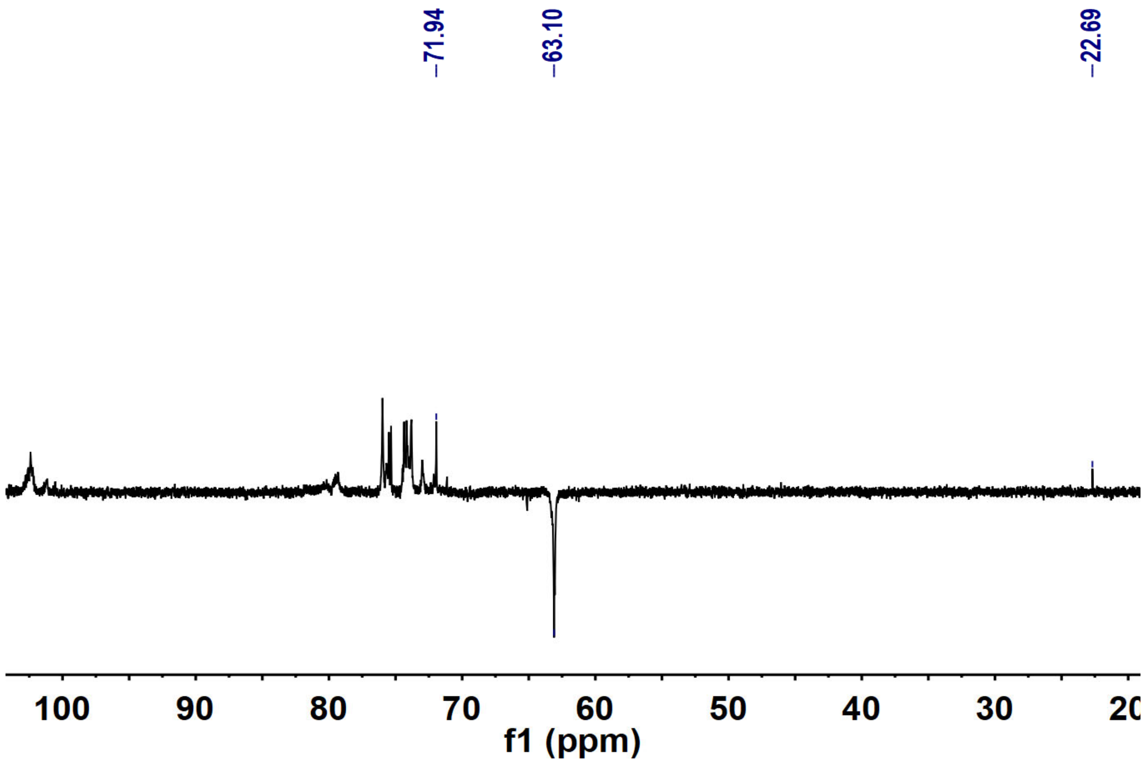

Figure S2. DEPT-135 spectrum of F2

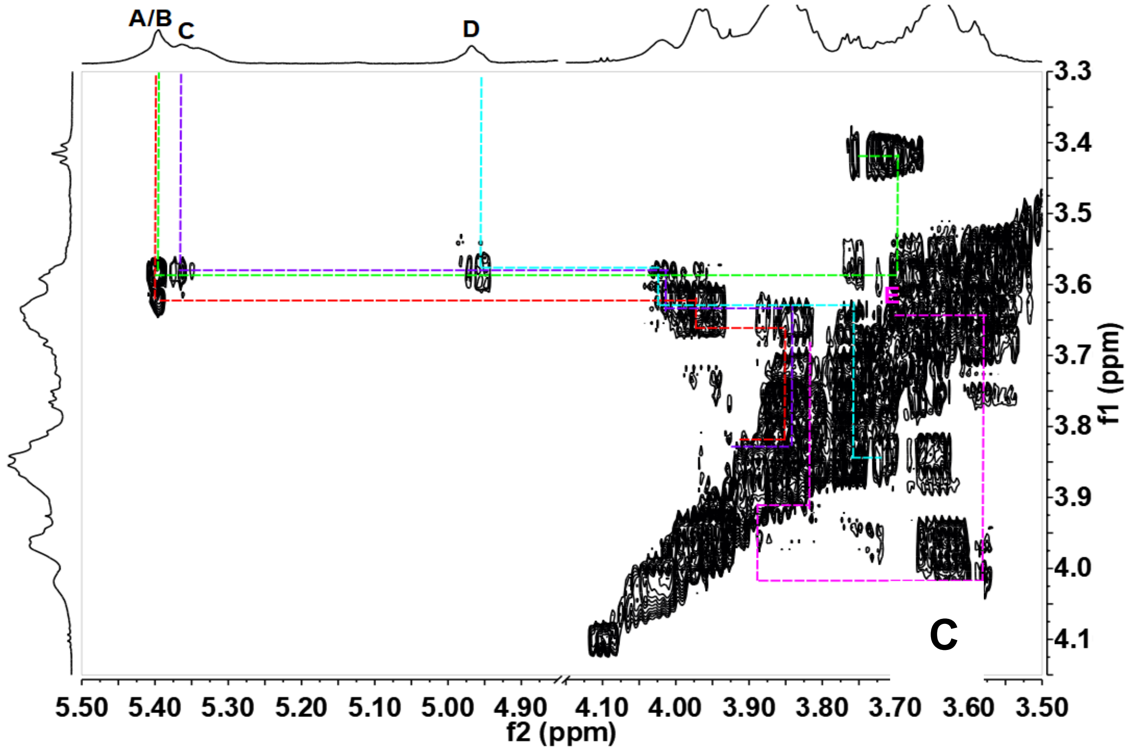

Figure S3. ^{1}H-^{1}H COSY spectrum of the F2

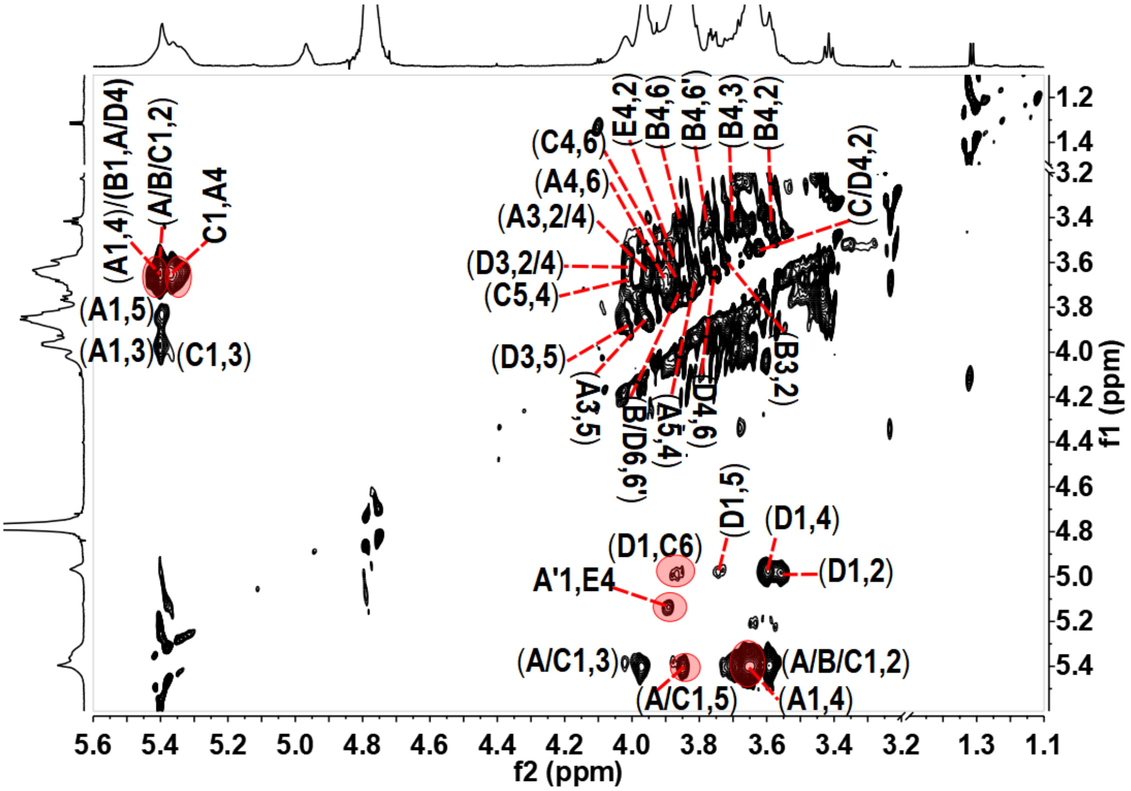

Figure S4. ^{1}H-^{1}H ROESY spectrum of the F2

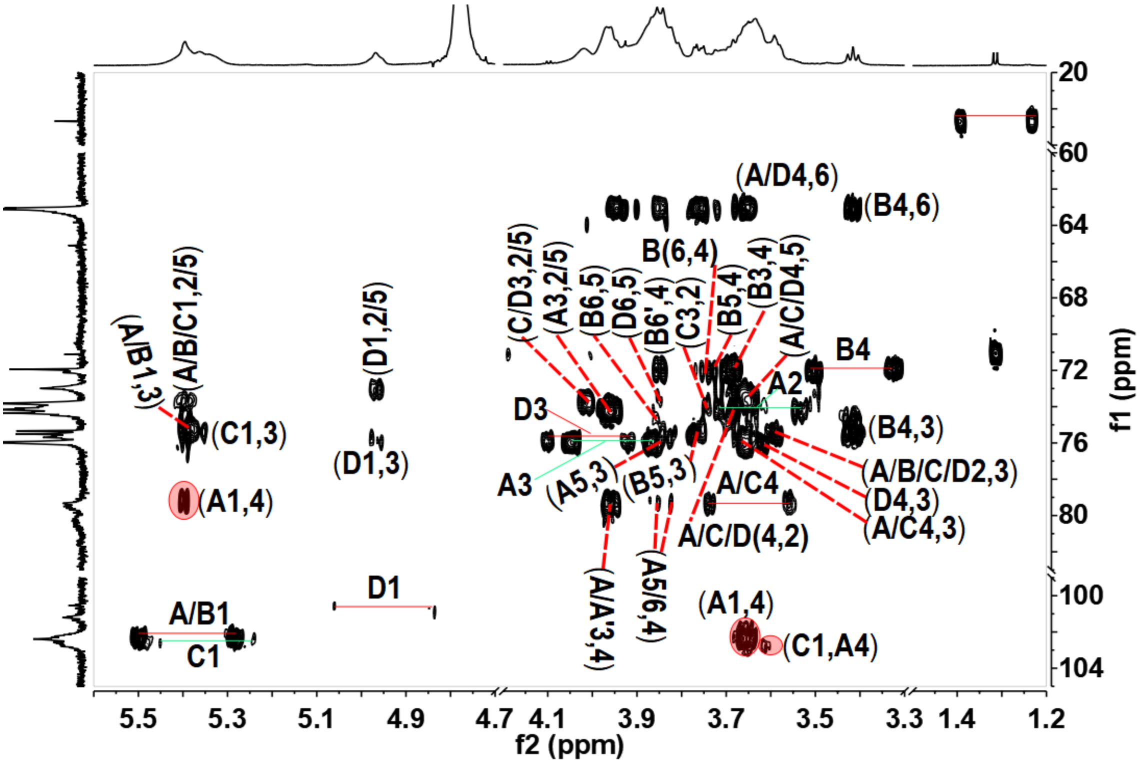

Figure S5. ^{1}H-^{13}C HMBC spectrum of F2

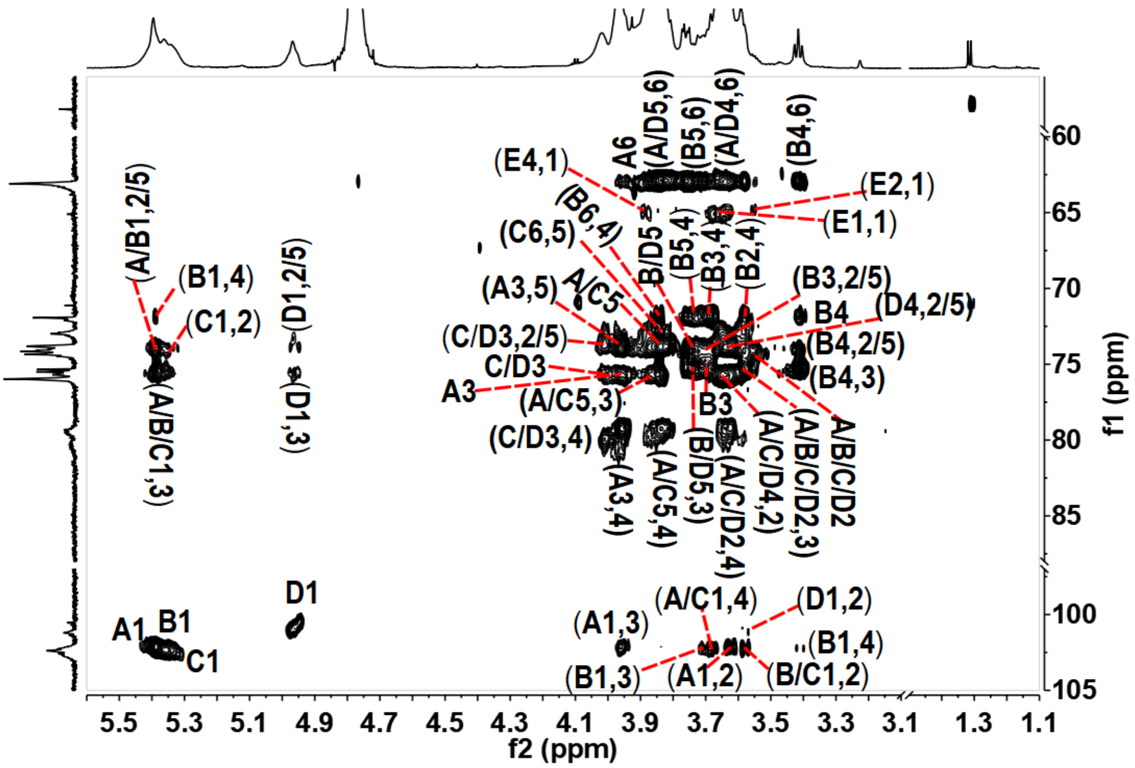

Figure S6. ^{1}H-^{13}C HSQC-TOCSY spectrum of F2.

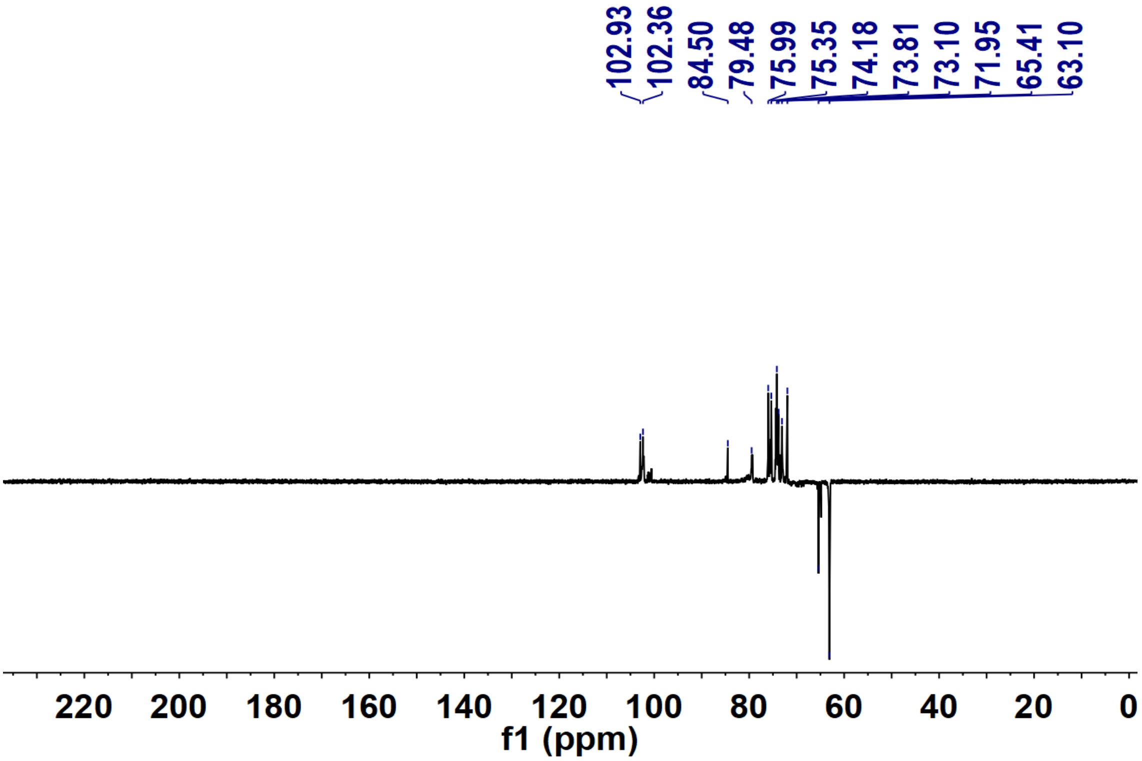

Figure S7. DEPT-135 spectrum of F3

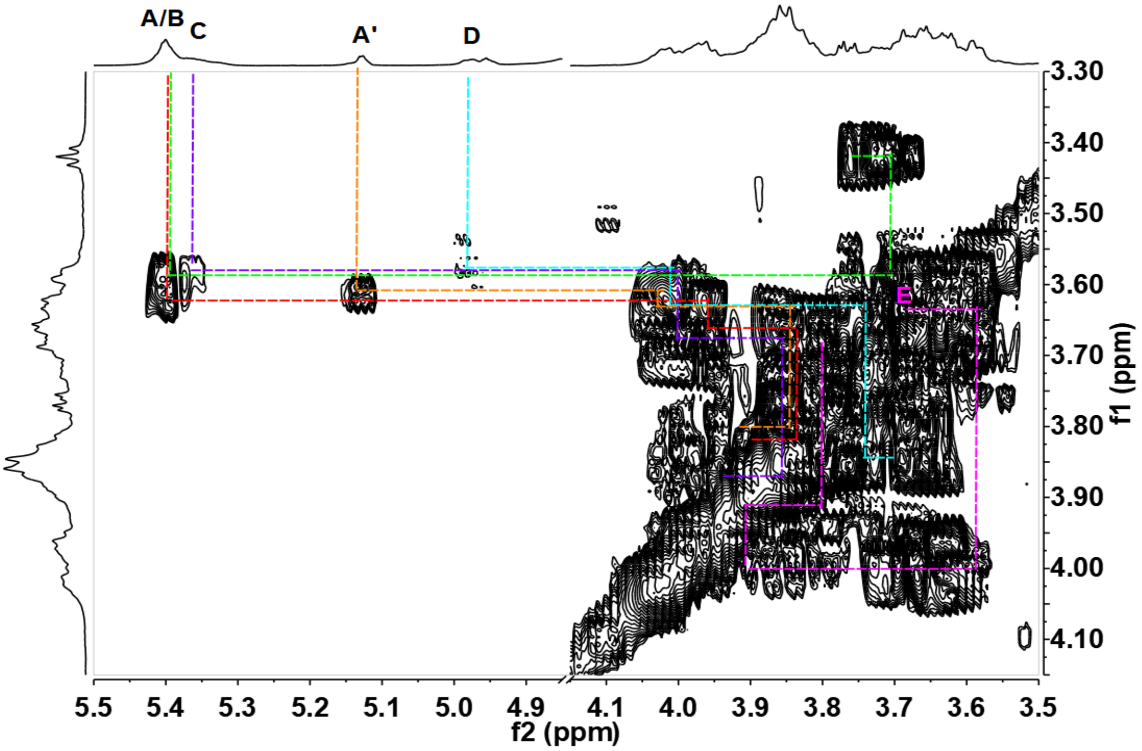

Figure S8. ^{1}H-^{1}H COSY spectrum of F3

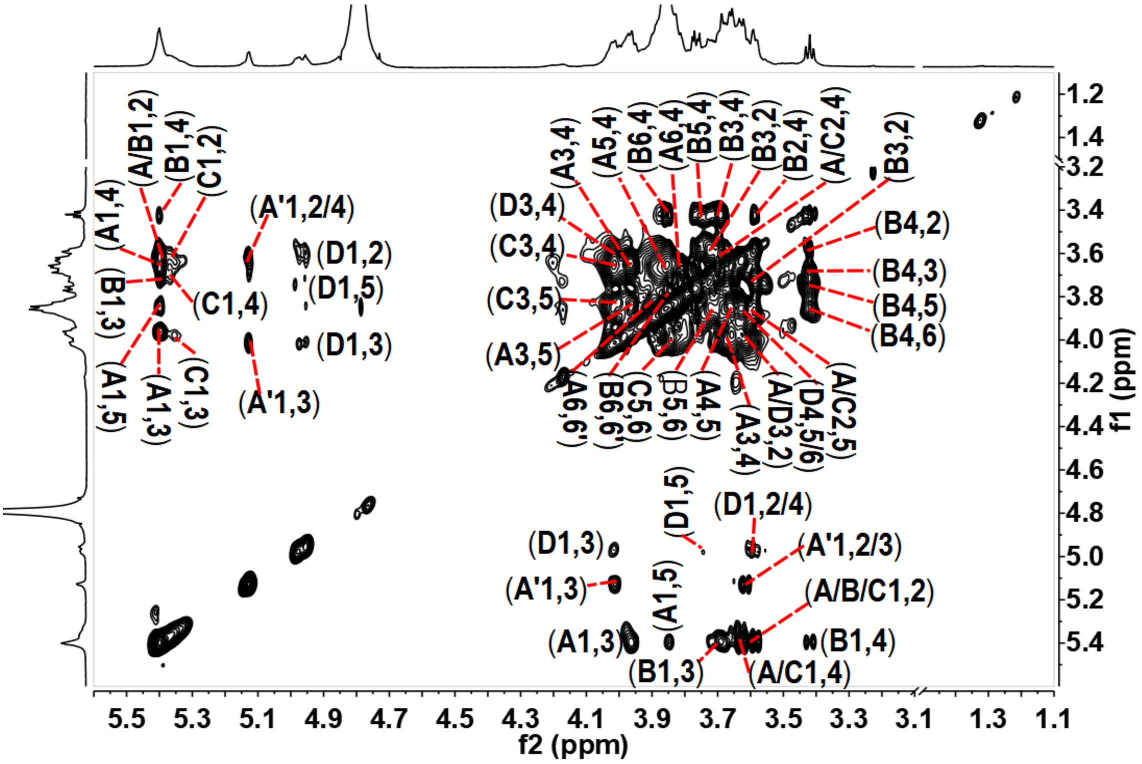

Figure S9. ^{1}H-^{1}H TOCSY spectrum of the F3

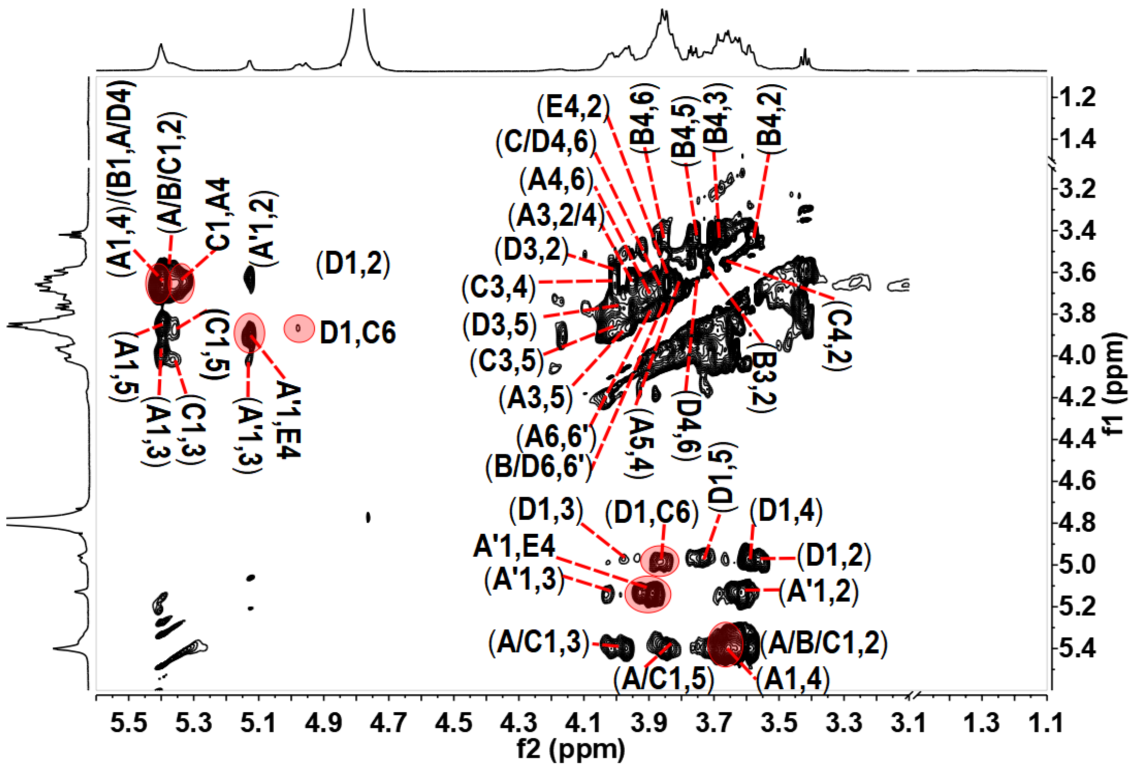

Figure S10. ^{1}H-^{1}H ROESY spectrum of the F3

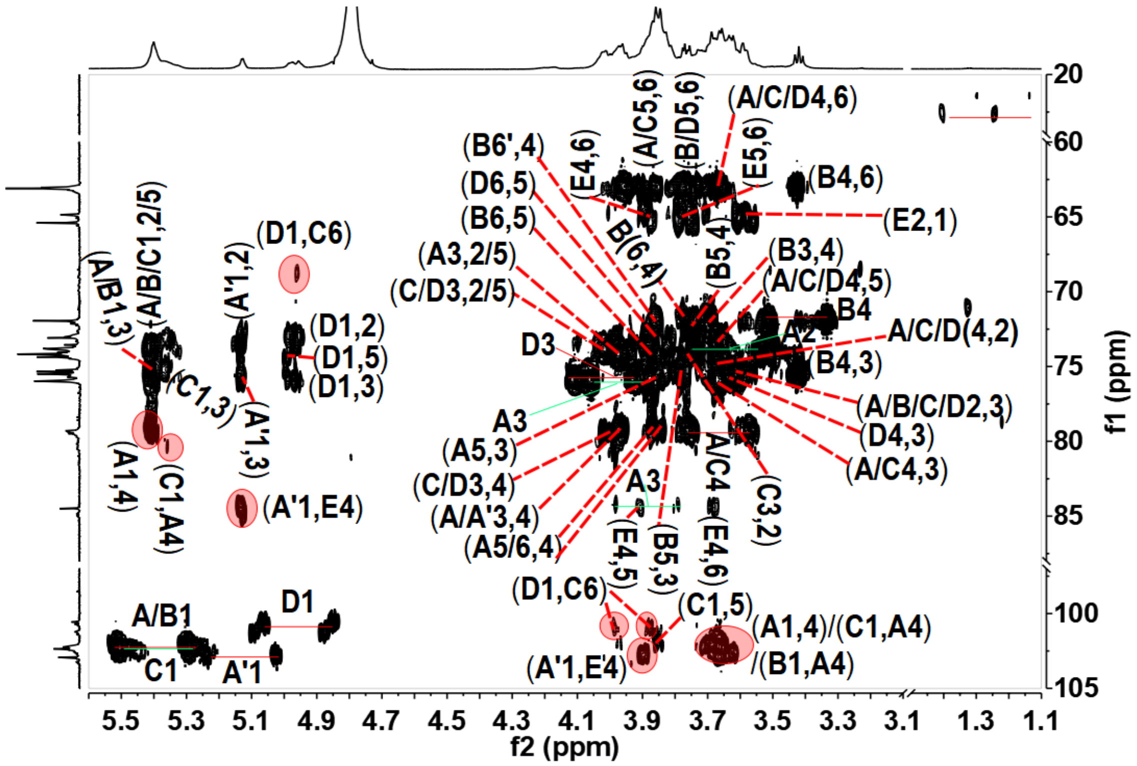

Figure S11. ^{1}H-^{13}C HMBC spectrum of the F3

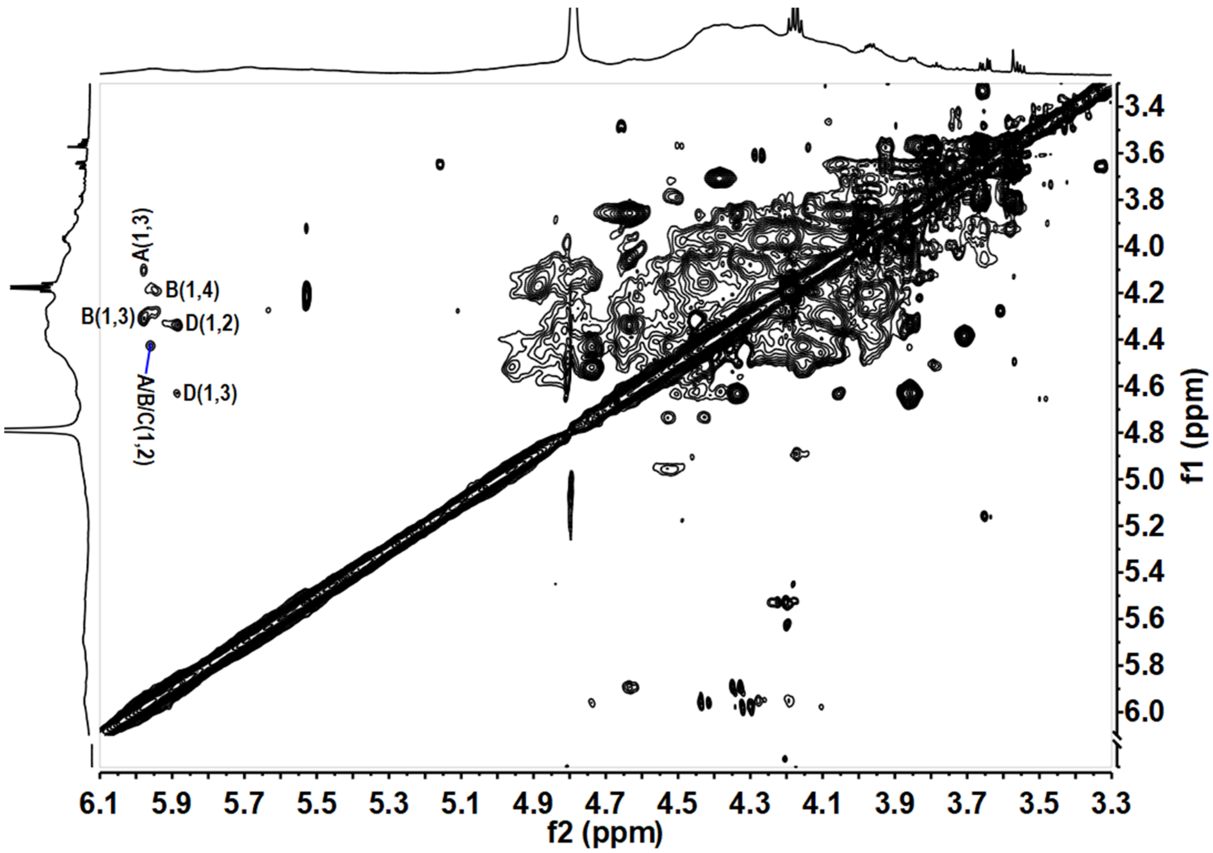

Figure S12. ^{1}H-^{1}H TOCSY spectrum of the TPGS

Table S1. ^{1}H and ^{13}C NMR chemical shifts of TPGS.

Residues	H/C	Chemical Shifts (δ, ppm)					
		1	2	3	4	5	6
**A**	H	5.98	4.45	4.63/4.07	**3.81**	3.79	4.32/4.42
	C	99.13	77.72	81.11/76.19	**79.91**	74.80	69.23
**B**	H	5.97	4.42	4.27	4.18	3.79	4.32/4.42
	C	99.26	77.78	79.91	73.61	74.80	69.23
**C**	H	5.93	4.40	4.63	**3.81**	3.79	**3.86/4.20**
	C	99.45	77.72	81.11	**79.91**	74.80	**70.55**
**D**	H	5.71	4.33	4.63	**3.81**	3.79	4.32/4.42
	C	96.74	78.11	81.11	**79.91**	74.80	69.23

[a] *Values in boldface indicate glycosylated positions.*
